# Effect of *Moringa stenopetala* leaf extracts on the physicochemical characteristics and sensory properties of lagered beer

**DOI:** 10.1002/fsn3.2672

**Published:** 2021-11-30

**Authors:** Gedefaw Mazengia, Engeda Dessalegn, Tilku Dessalegn

**Affiliations:** ^1^ BGI Ethiopia Addis Ababa Ethiopia; ^2^ Department of Chemistry Hawassa College of Education Hawassa Ethiopia; ^3^ School of Nutrition, Food Science and Technology Hawassa University Hawassa Ethiopia

**Keywords:** haziness, lagered beer, *Moringa stenopetala*, natural antioxidant

## Abstract

Flavor instability resulting from beer storage and oxidation is the most important quality‐related problem in the brewing industry. This study evaluated the influence of adding 80% ethanolic extract of *Moringa stenopetala* leaf to lagered beer at 400, 600, and 800 ppm concentrations for 30‐, 60‐, and 90‐day storage time at room temperature. The effect of physicochemical properties of the beer incorporated with leaf extract of *Moringa stenopetala* (LEMS) was evaluated using the American Society of Brewing Chemists method of analysis. Sensory acceptability of the beer treated with LEMS was evaluated using nine hedonic scales over a period of storage time. Original gravity (11.06–11.08), apparent extract (3.68–3.77), pH (4.23–4.40), vicinal diketone (0.07–0.09), and alcohol content (4.76–4.81) were not altered by the incorporation of LEMS at any level of treatment and over a period of storage time. The beer color (8.88–9.70 EBC), bitterness (13.62–15.56 bitterness unit), calcium ion (44.18–52.04 ppm), and foam stability (201.5–246.5) of beer increased with increasing LEMS concentration, but a significant haziness reduction (1.23–0.63) was observed. However, the storage time decreased both haziness and foam stability of LEMS‐incorporated beer. The incorporation of LEMS at an optimum level kept its quality for 90 days better than the usual antioxidant (potassium metabisulfite) added in beer. The sensory analysis also supported the beer treated with 600 ppm of LEMS as the best overall acceptability. The result indicates a promising use of LEMS as a functional ingredient in beer to reduce beer oxidation probability and keep its freshness for a period of storage time.

## Graphical abstract

This study evaluates the influence of adding the extract of *Moringa stenopetala* leaf to laagered beer at 400, 600, and 800 ppm concentrations for 30‐, 60‐, and 90‐day storage time at room temperature. The effect of extract on physicochemical properties was evaluated using the American Society of Brewing Chemists method of analysis. The storage time of beer had a significant effect on the quality of beer freshness, and the incorporation of LEMS at an optimum level kept its quality for a prominent time better than the usual antioxidant (potassium metabisulfite) added in beer.

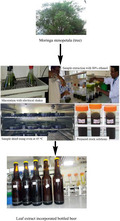

## 1 INTRODUCTION

Beer, the complex brewed beverage made from malt, hops, water, and yeast, is widely consumed all over the world for its fresh taste, low calories, and nutritional value (Pereira et al., 2020). It contains various compounds with antioxidant activity mainly originated from raw materials or formed during processing (Martinez‐Gomez et al., 2020). Lagered beer is a light‐colored highly carbonated type of beer. The term lager is used to denote the beer produced from bottom‐fermenting yeast. Flavor stability has become the most important factor in determining the shelf life of packed beer, and prolonging shelf life by delaying flavor staling is one of the greatest challenges faced by the brewer. Although the flavor stability of beer depends primarily on the oxygen content of the packaged beer, the brewing process and the raw materials used can influence the flavor stability. The antioxidant potential and bioactive compounds in beer decline with storage time, and quality loss of beer due to storage time affects consumer acceptability. Therefore, attention is now increasingly shifted toward increasing the antioxidant activity of beer itself or addition of natural antioxidant.

*Moringa* is one of the most powerful sources of natural antioxidants by supplying the free atoms and mitigating the effect of free radicals. It contains a high concentration of phenolic acids, flavonoid, and alkaloid compounds (Engeda & Vasantha, 2021; Nadeem et al., 2013). The accumulated antioxidant potential present in *Moringa stenopetala* leaf extract and its abundantly available bioactive compounds could be used in beer to keep its shelf life over the period of storage time. Besides the bioactive and prohealth properties, the beer phenolics also have a technological significance, since they play a crucial role in haze stability, foam maintenance, and physicochemical stability (Fumi et al., 2011). This research evaluated the effect of *Moringa stenopetala* leaf extract on the physicochemical properties of lagered beer during storage time.

## 2 MATERIALS AND METHODS

### 2.1 Raw material collection

The leaf of *Moringa stenopetala* was collected from the compound of Hawassa College of Education, where it was botanically classified and planted for research purpose by the institute. Malt was found from Assela malt factory through BGI Ethiopia Hawassa Brewery, and brewing liquor (water) treated for brewing standard was taken from Brasseries et glacières internationales (BGI) Ethiopia Hawassa Brewery. *Saccharomyces cerevisiae* yeast S‐189 type was collected from the newly propagated yeast in BGI Ethiopia at the secondary stage of fermentation through sterilized line and container.

### 2.2 Experimental design and treatments

Factorial design was used to investigate the effect of LEMS at 400, 600, and 800 ppm concentrations with 1‐, 30‐, 60‐, and 90‐day storage time on the physicochemical property and sensory evaluation of beer. The experiment had a positive control of a beer with usual 12 ppm of potassium metabisulfite (KMS) and a negative control of beer without antioxidant (control). The level of treatment was selected based on trial experiment on sensory evaluation where selected trained panelists evaluated the maximum possible amount of LEMS to be added without affecting the original beer taste (Alejandra, 2002). Based on the trial experiment, three levels of treatments including 400 ppm (S03), 600 ppm (S04), and 800 ppm (S05) were selected.

### 2.3 *Moringa* *stenopetala* leaf extract preparation

The leaves were collected carefully, wrapped up with aluminum foil, and taken to Hawassa University Food Science and Postharvest Technology Laboratory. It was then washed with distilled water, air‐dried under a shed, and grounded using an electrical grinder (Nadeem et al., 2013). The fine powder of leaf was mixed with 80% ethanol with 1 g to 10 ml ratio using a triplicate Pyrex beaker of 1000 ml. The beakers were tightly closed with a cover bush and macerated using an electrical shaker for 18 h. The residue was then separated and dried at 45°C using an oven drier. A stock solution was prepared and stored in refrigerator at 4°C for subsequent usage (Siddhuraju & Becke, 2003).

### 2.4 Beer preparation

The beer was prepared according to the method developed by Pires and Brányik (2015) using dry milling system. The mash was prepared from malt, which was milled into 2.25‐mm‐diameter sieve and mixed with water at 55°C with 2.3 L/kg water to grist ratio in commercial brewing plant. The mash was heated to 64°C with 20‐min rest time and 74°C with 15‐min rest time. After saccharification of the mash, the temperature was raised to 78°C, and the mash was filtered using the mash filter. The filtered wort was then boiled with 0.12 kg of CO_2_‐extracted hop per hectoliter for 60 min. Hot trub was separated using wort‐settling tank after 20‐min rest, and the hot wort was cold to 10°C and aerated to 18 ppm of oxygen. The wort was then left for primary fermentation using a conical fermenter vessel at 12°C until the original gravity decreased from 18 ^o^P to 8 ^o^P and secondary fermentation at 16°C until the vicinal diketones (VDK) reached <0.18 ppm. After completing the fermentation process, the beer was stored at −2°C and kept for 2‐day lagering period for maturation in the fermenter tank at 0.5 bar counter pressure using carbon dioxide. The matured beer was then purged and filtered using the candle filter with the help of filter aids. The filtered beer was diluted to 11.05 ^o^P using de‐aerated water and carbonated to 5.8 g/L CO_2_, which was then packed aseptically using 330‐ml sanitized amber bottles and crowned with manual crowner after the antioxidant dosing at different concentrations. All samples were labeled and pasteurized using a tunnel pasteurizer at 60°C for about 20 min and stored at room temperature. The physicochemical properties and sensory properties of each sample were analyzed in each 30 days for three consecutive months starting from the first day of sample preparation.

### 2.5 Physicochemical analysis

The physicochemical properties of lager beer such as pH, haziness, vicinal diketones, original extract, real extract, alcohol test, and foam stability was analyzed using the standard American Society of Brewing Chemists (ASCB) method of analysis. The vicinal diketones (VDK), color, and bitterness of each beer sample were measured using a spectrophotometer (GENESYS 1OS UV‐VIS) at 335, 430, and 275 nm absorbance, respectively (American Society of Brewing Chemists, 2009).

### 2.6 Calcium ion analysis

The concentration was determined according to ASBC (2009) by a method called complexometric titration. First, 10 ml sample was added into a conical flask and filled with distilled water to 100 ml. Then, 3 ml of potassium hydroxide solution was added and mixed well with magnetic stirrer. From the properly‐mixed solution, 1 pinch of Cal‐Red indicator was added and titrated with 0.01 mol/L of EDTA (ethylenediamine tetraacetic acid) to end point until the color changed from pink to gray blue. The volume used for titration was taken as V1. Finally, calcium ion concentration was calculated using the below formula:

Calcium (ppm) = 40.08 * *V*1 * 0.01 * 1000/ml of sample

### 2.7 Sensory properties

Sensory evaluation of the untreated beer, LEMS, and potassium metabisulfite–treated beer at different storage time was carried out by 15 well‐trained panelists selected from BGI Ethiopia Hawassa Brewery (American Society of Brewing Chemists, 2008). The interaction effect of LEMS and storage time on the color, foam stability, bitterness, mouth feeling, aroma, flavor, and overall acceptability of the beer was rated with nine hedonic scales from 1 (extremely dislike) to 9 (extremely like). Of the cooled sample at 4°C, 50 ml was served monadically, in glass cups codified with three‐digit numbers.

### 2.8 Statistical analysis

The experimental data were subjected to analysis of variance (ANOVA), and Duncan's multiple range tests were used to detect the difference (*p* ≤ .05) between the mean values. Statistical analyses were performed with the statistical program SAS 9.0 (SAS Inc.) and origin 8 software package, and the data were presented as mean and standard deviation.

## 3 RESULTS AND DISCUSSION

### 3.1 Effect of LEMS and storage time on the physicochemical properties of beer

The effect of LEMS and storage time on the physicochemical properties of lagered beer is stated in Table 1. The present study exhibits no significant differences (*p* > .05) among treatments on the original gravity, apparent extract, and VDK and alcohol content values. All those physical properties were found similar with variation of treatment, which might be happened due to the cease in fermentation. These parameters were linearly correlated with the fermentation process and were affected during the stage of fermentation in the fermenter vessel. According to Claudio et al. (2019), the VDK of beer was found with insignificant changes for more than 3 months after the cease of beer fermentation, and it is highly affected by fermentation process.

**TABLE 1:** Effect of LEMS and storage time on physicochemical profile of lagered beer

Storage time	Treatment	AE (^o^P)	Alcohol (V/V)	OG (^o^P)	Color (EBC)	pH	VDK (ppm)	Bitterness (BU)	Calcium (ppm)	Haziness (90°)	Foam stability
	S01	3.72 ± 0.02^a^	4.78 ± 0.03^a^	11.06 ± 0.07^a^	8.88 ± 0.04^d^	4.40 ± 0.05^a^	0.08 ± 0.12^a^	13.62 ± 0.19^d^	44.18 ± 0.11^d^	1.23 ± 0.04^d^	201.50 ± 1.62^g^
	S02	3.76 ± 0.01^a^	4.78 ± 0.02^a^	11.07 ± 0.07^a^	8.90 ± 0.07^d^	4.41 ± 0.04^a^	0.09 ± 0.31^a^	13.65 ± 0.07^d^	44.18 ± 0.06^d^	1.21 ± 0.05^dc^	210.10 ± 1.72^e^
D01	S03	3.68 ± 0.04^a^	4.81 ± 0.01^a^	11.07 ± 0.14^a^	9.48 ± 0.04^b^	4.42 ± 0.14^a^	0.07 ± 0.31^a^	15.05 ± 0.07^c^	48.04 ± 0.09^c^	0.88 ± 0.07^e^	228.50 ± 1.12^c^
	S04	3.74 ± 0.02^a^	4.81 ± 0.01^a^	11.07 ± 0.07^a^	9.59 ± 0.07^b^	4.40 ± 0.14^a^	0.08 ± 0.01^a^	15.38 ± 0.04^b^	49.17 ± 0.02^b^	0.68 ± 0.03^g^	239.10 ± 1.54^b^
	S05	3.77 ± 0.01^a^	4.80 ± 0.05^a^	11.08 ± 0.07^a^	9.70 ± 0.07^a^	4.39 ± 0.12^a^	0.09 ± 0.12^a^	15.56 ± 0.05^a^	52.04 ± 0.09^a^	0.63 ± 0.04^g^	246.50 ± 1.95^a^
	S01	3.73 ± 0.04^a^	4.78 ± 0.02^a^	11.07 ± 0.42^a^	8.92 ± 0.05^d^	4.39 ± 0.11^a^	0.08 ± 0.11^a^	13.60 ± 0.03^d^	44.15 ± 0.07^d^	1.25 ± 0.03^c^	197.00 ± 1.41^h^
	S02	3.73 ± 0.01a	4.77 ± 0.01^a^	11.06 ± 0.07^a^	8.93 ± 0.09^d^	4.41 ± 0.08^a^	0.08 ± 0.03^a^	13.63 ± 0.11^d^	44.17 ± 0.06^d^	1.19 ± 0.04^d^	210.30 ± 2.12^e^
D30	S03	3.69 ± 0.01a	4.81 ± 0.06^a^	11.07 ± 0.07^a^	9.48 ± 0.11^b^	4.42 ± 0.03^a^	0.09 ± 0.07^a^	14.98 ± 0.05^c^	48.08 ± 0.06^c^	0.85 ± 0.06^e^	228.40 ± 1.66^c^
	S04	3.74 ± 0.04^a^	4.80 ± 0.04^a^	11.06 ± 0.08^a^	9.60 ± 0.05^b^	4.41 ± 0.02^a^	0.09 ± 0.01^a^	15.36 ± 0.09^b^	49.15 ± 0.07^b^	0.60 ± 0.04^g^	238.90 ± 1.83^b^
	S05	3.74 ± 0.01a	4.79 ± 0.04^a^	11.08 ± 0.07^a^	9.73 ± 0.04^a^	4.39 ± 0.02^a^	0.09 ± 0.08^a^	15.56 ± 0.06^a^	52.05 ± 0.43^a^	0.40 ± 0.04^h^	246.39 ± 2.24^a^
	S01	3.73 ± 0.03a	4.78 ± 0.06^a^	11.06 ± 0.08^a^	9.38 ± 0.06^c^	4.30 ± 0.03^b^	0.08 ± 0.08^a^	13.60 ± 0.07^d^	44.13 ± 0.08^d^	1.38 ± 0.03^b^	192.00 ± 1.83^i^
	S02	3.73 ± 0.06^a^	4.77 ± 0.03^a^	11.07 ± 0.08^a^	9.08 ± 0.08^cb^	4.42 ± 0.04^a^	0.08 ± 0.02^a^	13.59 ± 0.07^d^	44.17 ± 0.06^d^	1.25 ± 0.07^c^	204.95 ± 2.12^f^
D60	S03	3.71 ± 0.02^a^	4.80 ± 0.04^a^	11.08 ± 0.06^a^	9.50 ± 0.12^b^	4.41 ± 0.08^a^	0.09 ± 0.03^a^	14.94 ± 0.05^c^	48.07 ± 0.13^c^	0.75 ± 0.07^f^	227.95 ± 1.77^c^
	S04	3.72 ± 0.03^a^	4.80 ± 0.02^a^	11.06 ± 0.05^a^	9.61 ± 0.03^b^	4.39 ± 0.01^a^	0.09 ± 0.04^a^	15.36 ± 0.03^C^	49.12 ± 0.07^b^	0.61 ± 0.04^g^	238.10 ± 1.41^b^
	S05	3.73 ± 0.04^a^	4.79 ± 0.01^a^	11.08 ± 0.05^a^	9.72 ± 0.04^a^	4.40 ± 0.02^a^	0.08 ± 0.06^a^	15.62 ± 0.03^a^	52.04 ± 0.22^a^	0.41 ± 0.05^h^	246.10 ± 2.24^a^
	S01	3.73 ± 0.04^a^	4.79 ± 0.02^a^	11.08 ± 0.07^a^	9.55 ± 0.14^cb^	4.23 ± 0.01^c^	0.08 ± 0.01^a^	13.58 ± 0.04^d^	44.17 ± 0.07^d^	1.51 ± 0.01^a^	186.40 ± 2.19^j^
	S02	3.72 ± 0.05^a^	4.76 ± 0.04^a^	11.07 ± 0.04^a^	9.47 ± 0.05^b^	4.34 ± 0.02^a^	0.08 ± 0.03^a^	13.57 ± 0.06^d^	44.17 ± 0.09^d^	1.36 ± 0.02^b^	198.50 ± 1.54^gh^
D90	S03	3.71 ± 0.04^a^	4.78 ± 0.03^a^	11.08 ± 0.07^a^	9.54 ± 0.04^b^	4.37 ± 0.02^a^	0.09 ± 0.06^a^	14.96 ± 0.04^c^	48.15 ± 0.06^c^	0.81 ± 0.01^fe^	225.40 ± 2.12^d^
	S04	3.73 ± 0.01^a^	4.80 ± 0.01^a^	11.07 ± 0.08^a^	9.60 ± 0.03^b^	4.38 ± 0.01^a^	0.09 ± 0.07^a^	15.33 ± 0.01^b^	49.16 ± 0.06^b^	0.64 ± 0.02^g^	237.60 ± 2.24^b^
	S05	3.73 ± 0.07^a^	4.79 ± 0.05^a^	11.08 ± 0.02^a^	9.74 ± 0.03^a^	4.39 ± 0.01^a^	0.09 ± 0.06^a^	15.59 ± 0.05^a^	52.04 ± 0.25^a^	0.44 ± 0.01^h^	245.90 ± 1.71^b^

Note

Values are mean and standard deviation of duplicated determinations; the mean values with the same letter across the column are not significantly different at *p* ˂ .05. Numbers written after ‘S’ represented the concentration of antioxidants, 01 is with no antioxidant, 02 is with 12 ppm of KMS, 03 is 400 ppm of LEMS, 04 is with 600 ppm of LEMS, and 05 is 800 ppm of LEMS.

Abbreviations: AE, apparent extract; OG, original gravity.

The result was also supported by the research conducted by Ulloa et al. (2017), where the addition of propolis extract in lagered beer did not alter the original gravity, apparent extract, VDK, and alcohol content values. Likewise, we did not find any significant correlation between treatments and storage time on the original gravity, apparent extract, VDK, and alcohol content. According to the study conducted by Stephenson and Bamforth (2002), the addition of antioxidants and storage time affects the beer aroma and flavor without a significant effect on the original extract and its alcohol content. The proximate analysis of methanolic leaf extracts of *Moringa stenopetala* by Debebe and Eyobel (2017) proved that LEMS with limited concentration contributes an insignificant amount of sugar.

The pH values of each treatment were found within the desirable parameters, generally between 4.23 and 4.42, protecting the product against pathogens (Suzuki et al., 2006). The addition of LEMS causes no significant pH alteration at each level of treatment, which might be due to the interference of antioxidants from hydrogen ion generation (Perron & Brumaghim, 2009). Nonetheless, the pH was significantly affected by storage time for the control sample without antioxidant starting from the second month of storage. There was a reduction in pH from 4.40 to 4.23, which could be from the oxidation of beer that might generate weak acids such as acetic acid, which is supported by Bamforth et al. (2018) who found an excessive production of acetic acid from a beer stored with limited antioxidant that reduces the pH level after 2 months of storage from 4.43 to 4.32.

Beer color enhancement was observed with increasing concentration of LEMS in each level of treatment. It was increased significantly from 8.88 EBC for the untreated beer to 9.70 EBC (Table 1) for the maximum concentration of LEMS used. Indeed, there was no visible observed difference between the controlled beer without antioxidant and the beer treated with potassium metabisulfite. This color enhancement might be due to increasing the concentration of the pigment of LEMS. This result supported the research held by He et al. (2012) that *Moringa stenopetala* contains a significant number of carotenoids, which enables significant enhancement of beer color. This could help the modern breweries to reduce their caramel consumption used for color enhancement at the brew house.

There was a significant increment in the color of the untreated beer with an increase in storage time from 8.88 to 9.55 EBC that could arise from the oxidation of beer. According to the findings of Vanderhaegen et al. (2006) on the chemistry of beer aging, oxidation made a significant impact on a beer color. On the contrary, we did not observe significant color variation due to storage time along the LEMS‐treated samples (Table 1), but there was little color increment for potassium metabisulfite‐treated beer after the second month of storage, which might be correlated with its antioxidant potential strength as compared to LEMS.

The addition of LEMS on lagered beer had significantly increased (Table 2) the bitterness unit (BU) from 13.62 (control sample) to 15.56 (sample treated with 800 ppm of LEMS). Increasing the concentration of LEMS from 400 to 800 ppm raised the bitterness level from 15.05 BU to 15.56 BU. The beer sample treated with potassium metabisulfite had no significant difference (*p* > .05) when compared with the control. The increasing effect of bitterness with the addition of LEMS might be due to the available alpha acids in the LEMS. According to the study conducted by Shih et al. (2011), *Moringa* leaf had a significant amount of bittering compounds called alpha acids, which is in line with the present finding. Apparently, no noticeable change in bitterness in each level of treatment along the storage time was observed.

**TABLE 2:** The effect of LEMS and storage time on sensory properties of beer

Storage time (days)	Treatment	Foam stability	Beer color	Bitterness	Beer body	Flavor and aroma	Overall acceptability
	S01	7.80 ± 0.07^d^	8.28 ± 0.03^c^	8.23 ± 0.04^c^	8.23 ± 0.03^d^	8.54 ± 0.04^c^	8.23 ± 0.05^d^
	S02	7.88 ± 0.04^d^	8.28 ± 0.04^c^	8.19 ± 0.05^c^	8.33 ± 0.04^dc^	8.53 ± 0.13^c^	8.28 ± 0.04^de^
Day 1	S03	8.28 ± 0.05^c^	8.35 ± 0.07^c^	8.48 ± 0.09^b^	8.63 ± 0.03^b^	8.67 ± 0.03^b^	8.58 ± 0.04^ba^
	S04	8.43 ± 0.04^b^	8.55 ± 0.07^b^	8.55 ± 0.12^a^	8.65 ± 0.04^b^	8.70 ± 0.08^a^	8.68 ± 0.02^a^
	S05	8.63 ± 0.03^a^	8.05 ± 0.07^fe^	8,10 ± 0.03^d^	8.73 ± 0.04^a^	8.38 ± 0.04^d^	8.15 ± 0.02^e^
	S01	7.73 ± 0.07^e^	8.16 ± 0.09^e^	8.19 ± 0.07^b^	8.25 ± 0.07^d^	8.11 ± 0.04^e^	8.01 ± 0.02^f^
	S02	7.85 ± 0.07^d^	8.25 ± 0.07^d^	8.20 ± 0.07^c^	8.35 ± 0.09^dc^	8.50 ± 0.07^c^	8.26 ± 0.04^d^
Day 30	S03	8.40 ± 0.08^b^	8.35 ± 0.11^c^	8.47 ± 0.05^c^	8.65 ± 0.08^b^	8.66 ± 0.03^b^	8.56 ± 0.01^ba^
	S04	8.53 ± 0.04^ba^	8.55 ± 0.07^b^	8.54 ± 0.09^b^	8.75 ± 0.06^a^	8.71 ± 0.04^a^	8.67 ± 0.03^a^
	S05	8.58 ± 0.02^ba^	8.08 ± 0.08^fe^	8,11 ± 0.07^d^	8.73 ± 0.04^a^	8.40 ± 0.07^d^	8.16 ± 0.08^e^
	S01	7.23 ± 0.05^f^	7.40 ± 0.07^g^	8.24 ± 0.04^c^	8.25 ± 0.12^d^	7.51 ± 0.09^f^	7.57 ± 0.05^g^
	S02	7.69 ± 0.01^e^	8.23 ± 0.08^d^	8.21 ± 0.07^c^	8.28 ± 0.04^d^	8.51 ± 0.07^c^	8.19 ± 0.03^d^
Day 60	S03	8.25 ± 0.06^c^	8.23 ± 0.04^d^	8.47 ± 0.04^b^	8.63 ± 0.03^b^	8.65 ± 0.08^b^	8.51 ± 0.06^b^
	S04	8.53 ± 0.04^b^	8.53 ± 0.03^b^	8.56 ± 0.07^a^	8.74 ± 0.07^a^	8.73 ± 0.06^a^	8.65 ± 0.01^a^
	S05	8.48 ± 0.04^b^	8.08 ± 0.07^fe^	8.10 ± 0.08^d^	8.72 ± 0.08^a^	8.37 ± 0.07^d^	8.14 ± 0.09^e^
	S01	6.28 ± 0.11^h^	6.75 ± 0.07^h^	8.19 ± 0.05^c^	7.98 ± 0.10^e^	6.85 ± 0.01^g^	6.66 ± 0.01^h^
	S02	7.08 ± 0.11^g^	7.98 ± 0.10^f^	8.20 ± 0.04^c^	8.23 ± 0.03^d^	8.10 ± 0.07^e^	8.03 ± 0.04^f^
Day 90	S03	8.23 ± 0.04^c^	8.20 ± 0.04^d^	8.49 ± 0.07^b^	8.50 ± 0.01^c^	8.62 ± 0.07^b^	8.30 ± 0.04^c^
	S04	8.35 ± 0.09^cb^	8.45 ± 0.03^cb^	8.54 ± 0.06^a^	8.54 ± 0.07^c^	8.65 ± 0.08^b^	8.48 ± 0.20^b^
	S05	8.43 ± 0.08^b^	8.15 ± 0.02^e^	8.13 ± 0.02^d^	8.65 ± 0.05^b^	8.40 ± 0.09^d^	8,11 ± 0.05^e^

Note

Values are mean and standard deviation of duplicated determinations; the mean values with the same letter across the column are not significantly different at *p* ˂ .05. Numbers written after ‘S’ represented the concentration of antioxidants, 01 is with no antioxidant, 02 is with 12 ppm of KMS, 03 is with 400 ppm of LEMS, 04 is with 600 ppm of LEMS, and 05 is 800 ppm of LEMS.

Increasing the concentration of LEMS in lagered beer significantly raised calcium ion concentration (Table 1) from 44.18 ppm of the untreated beer sample to 52.04 ppm of the beer treated with 800 ppm of LEMS. The increasing level of calcium ion concentration with increase in LEMS would be happened due to the higher content of calcium in *Moringa stenopetala* leaf. (Charles et al., 2011).

Regarding storage time, there was no observed significant difference on the calcium ion concentration of the lagered beer treated with LEMS. A study on beer aging by Vanderhaegen et al. (2006) demonstrated that the presence of antioxidants reduces the possible oxidation of metallic ions with reactive oxygen species. This could keep the calcium ion content of beer during storage, in the presence of antioxidants.

The haziness of the lagered beer treated with LEMS was highly affected by the concentration of LEMS (Table 1). It was reduced from 1.23 (the untreated beer sample) to 0.63 (the beer treated with 800 ppm of LEMS). This reduction in haziness might be happened due to the coagulation effect of LEMS as described by Aderinola et al. (2018) that LEMS had a high coagulation effect for water purification. The reduction in haziness with increasing concentration of LEMS in lagered beer might be due to its inhibition potential that reduces active protein suspensions, which are the precursor of beer haziness. On the contrary, potassium metabisulfite–treated beer had no significant effect on haziness when compared with the untreated beer.

Storage time did not reveal a change in haziness for LEMS‐treated beer sample at any level of concentration that might be due to the chemical stability of beer due to the incorporation of LEMS binds the possible reaction between phenolic compounds and proteins to create a turbid matter called haziness, but there was a significant increment of haziness for the untreated and potassium metabisulfite–treated beer samples from 1.23 to 1.51 and 1.21 to 1.51, respectively, due to storage time. The raise of haziness in those beer samples would have been the result of the reaction occurred between proteins and phenolic compounds. The finding on haziness of lagered beer is supported by Delvaux et al. (2000) where phenolic compounds and proteins primarily form visible haze in an aged beer up to 1.53 that its haziness increased by 23% from the date of production.

The effect of haziness due to the incorporation of LEMS (45% improvement for 600 ppm) is very impressive when compared with other beer stabilization agents. According to Delvaux et al. (2000), the addition of silica improves haziness by 15% and polyvinylpolypyrrolidone by 19%, which are still not comparable with LEMS efficiency on colloidal stability. Moreover, colloidal stabilization of beer with the above‐mentioned chemicals would affect customer health, and they are not economical according to the above findings, which highlight the potential interest to natural antioxidants such as LEMS.

The foam stability of beer is one of the basic quality indexes, which should be optimized in modern brewing technology to attain the global market. This finding verified that the addition of LEMS significantly increased (*p* < .05) the foam stability from 201.50 of the untreated beer to 246.50 of 800 ppm of LEMS‐treated beer (Table 1). This is because LEMS had enough foam‐promoting agents called polypeptides and iso‐α‐acids and deficient in foam‐negative materials called lipid‐binding proteins (Aderinola et al., 2018).

The foam stability of LEMS‐treated beer sample was not affected by storage time, which might be due to the availability of foam‐enhancing components due to the stabilizing power of LEMS. As per the study conducted by Bamforth and Kanauchi (2003), the beer foam stability depends on the interaction effect of several components, mainly proteins and polypeptides originated from malt and iso‐α‐acids. Accordingly, foam‐forming proteins and the available phenolic compounds from LEMS (Engeda & Vasantha, 2021) could keep its foam stability along a period of storage time. According to the study conducted by Evans et al. (2016), the proteins Z4, LTP1, hordoindoline/puroindoline, and hordeins, which are found abundantly in *Moringa stenopetala* leaf, have been associated with improved foam quality of beer.

Foam stability analysis for the untreated beer sample was found with significant reduction in the foam stability from 201.50 to 186.40. This could be due to the reduction in the concentration of total phenolic content happened by auto‐oxidation of beer over a storage time. However, it has been hypothesized that the major cause or prolonged survival of protein foams is high surface viscosity, which is due to the cohesion between molecules in the bubble wall and which can be estimated by oscillating disks. Such interactions may be between surface‐active molecules themselves or may be through the cross‐linking action of non‐surface‐active substance. Alternatively, high surface viscosity results of the diffusion coefficient of the surface‐active material are low, as may be the case with very high molecular weight proteins. The surface viscosity of mixed solutions will increase with respect to time, depending upon the rate at which surface interactions occur. The complex physicochemical principles underlying foam formation and structure will greatly help the brewer in achieving stable heads on beers. This surface interaction between surface‐active materials will be affected by storage time and shelf life of beer (Bamforth & Kanauchi, 2003).

### 3.2 Effect of LEMS and storage time on sensory properties of beer

The sensory analysis result of beer enriched with LEMS over a period of storage time is illustrated in Table 2. The foam stability acceptance of the untreated beer sample was less than the beer samples enriched with LEMS from 7.80 (like very much) to 8.63 (like extremely), and increasing in concentration of LEMS showed a significant increment in the acceptability of foam stability. The untreated beer sample foam acceptability decreased expressively from 7.8 (like very much) at the day of packaging to 6.28 (like slightly) after 3 months of storage. Nevertheless, the beer treated with different concentration of LEMS showed a slight change in consumers’ foam stability acceptance compared with the untreated beer sample from 8.63 (like extremely) at the date of packaging to 8.43 (like very much) after 3 months of storage for the 800 ppm of LEMS‐enriched beer sample.

The sensory acceptability result was found coherent with physicochemical analysis result, and the addition of LEMS increased significantly the acceptability of beer color due to the leaf pigment extracted (carotenoids) during the ethanolic extraction of *Moringa* (He et al., 2012). Beer color preference was increased from 8.28 (like very much) for the untreated sample to 8.55 (like extremely) for the sample with 600 ppm of LEMS. Increasing the concentration of LEMS above 600 ppm, however, decreased the acceptability of the beer due to color variation from pure golden to greenish golden rated to 8.08 (like very much).

The acceptability of color of beer without treatment decreased significantly from 8.28 (like very much) on the date of packaging to 6.75 (like moderately) after 90 days of storage, which might be due to the expected oxidation that made the beer to be dark and turbid. However, the beer treated with LEMS had no significant difference along the storage time, which might be correlated with low level of oxidation due to LEMS as compared to the untreated beer.

The beer bitterness is also supported by the physicochemical property analysis result in which the addition of more LEMS increased the bitterness. The beer sample enriched with 600 ppm of LEMS was preferred as the best bitterness level (8.55) while increasing the concentration of LEMS above 600 ppm decreased the acceptability due to the increase in bitterness level (8.10). Storage time did not affect the bitterness level acceptability of the beer treated with LEMS and the control beer. The addition of LEMS significantly increased beer body from 8.23 (like very much) for the untreated beer to 8.73 (like extremely) for the 800 ppm of LEMS‐treated beer while storage time had no significant effect on the beer body of each sample beer.

Flavor and aroma of the beer was affected by the addition of LEMS significantly. The sensory result showed that the addition of LEMS up to 600 ppm had better flavor than the untreated beer. However, increasing the concentration of the extract to 800 ppm decreased the flavor and aroma of the beer. The best flavor and aroma 8.7 (like extremely) was recorded for the sample treated with 600 ppm of LEMS. Increasing the concentration to 800 ppm of LEMS, decreased the aroma and flavor of the beer to 8.38 (like very much). Storage time significantly affected the untreated beer sample aroma and flavor from 8.54 (like extremely) at the date of production to 6.85 (like moderately) after 3 months of storage, but the storage time did not affect the aroma and flavor of LEMS‐treated beer at any level of treatment.

The overall acceptability of the beer samples had a significant difference by the addition of LEMS. The beer treated with 600 ppm of LEMS had the best overall acceptability when compared with the other samples. Storage time had a significant effect on the overall acceptability for the untreated beer in which the rate decreased from 8.23 (like very much) at the day of packaging to 6.66 (like moderately) after 3 months. Meanwhile, the overall acceptability did not change significantly for LEMS‐treated beer for three consecutive months. The beer sample treated with potassium metabisulfite changed its quality starting from the second month of storage as per the overall acceptability profile of the taste conducted.

## 4 CONCLUSION

Leaf extract of *Moringa stenopetala* could reduce lagered beer oxidation by keeping the basic physicochemical and freshness of beer, which could help breweries to afford fresh beer with long‐time storage. The optimum quantity of LEMS up to 600 ppm addition in the filtered beer exhibited the potential of reducing oxidation and kept beer freshness up to 3 months with the best mouth feeling. Moreover, LEMS improved the foam stability and haziness of filtered beer that raises the customer preference. It could be used as a fining agent for breweries to reduce beer haziness at a significant level. Original gravity, apparent extract, pH, and alcohol content were not affected by LEMS incorporation at any level in this study, which can be concluded that the usage of moderate concentration of LEMS in lagered beer has no effect on sensitive beer parameters that are corrected only in brew house. On the contrary, reduction in haziness and improvement in bitterness, color, foam stability, and mineral contents (calcium ion) could help the modern breweries to reduce worries of beer instability due to prolonged storage time (up to 90 days).

## CONFLICT OF INTEREST

The authors declare no conflict of interest.

## ETHICAL APPROVAL

Ethics approval was not required for this research, while sensory evaluation of beer consent was taken from the panelist.

## Data Availability

The data that support the findings of this study are available from the corresponding author upon reasonable request.
